# Effect of vertically oriented few-layer graphene on the wettability and interfacial reactions of the AgCuTi-SiO_2f_/SiO_2_ system

**DOI:** 10.1038/s41598-017-00295-5

**Published:** 2017-03-22

**Authors:** Z. Sun, L. X. Zhang, J. L. Qi, Z. H. Zhang, T. D. Hao, J. C. Feng

**Affiliations:** State Key Laboratory of Advanced Welding and Joining, Harbin Institute of Technology, Harbin, 150001 China

## Abstract

With the aim of expanding their applications, particularly when joining metals, a simple but effective method is reported whereby the surface chemical reactivity of SiO_2f_/SiO_2_ (SiO_2f_/SiO_2_ stands for silica fibre reinforced silica based composite materials and f is short for fibre) composites with vertically oriented few-layer graphene (VFG, 3–10 atomic layers of graphene vertically oriented to the substrate) can be tailored. VFG was uniformly grown on the surface of a SiO_2f_/SiO_2_ composite by using plasma enhanced chemical vapour deposition (PECVD). The wetting experiments were conducted by placing small pieces of AgCuTi alloy foil on SiO_2f_/SiO_2_ composites with and without VFG decoration. It was demonstrated that the contact angle dropped from 120° (without VFG decoration) to 50° (with VFG decoration) when the holding time was 10 min. The interfacial reaction layer in SiO_2f_/SiO_2_ composites with VFG decoration became continuous without any unfilled gaps compared with the composites without VFG decoration. High-resolution transmission electron microscopy (HRTEM) was employed to investigate the interaction between VFG and Ti from the AgCuTi alloy. The results showed that VFG possessed high chemical reactivity and could easily react with Ti even at room temperature. Finally, a mechanism of how VFG promoted the wetting of the SiO_2f_/SiO_2_ composite by the AgCuTi alloy is proposed and thoroughly discussed.

## Introduction

The wetting of solid metals and ceramics by liquid metals has attracted intensive interest due to its critical role in material processing, such as composite preparation, soldering and brazing. In general, ceramics are difficult to wet using traditional liquid alloys because of their high chemical stability^1^. In the past decade, great efforts have been made in exploring the wetting of alumina by a variety of liquid alloys, including Cu-Ti alloys^2, 3^, aluminium^4, 5^, a Ni-Nb interlayer^6^ and AgCuTi alloys^7–9^. However, the wettability of silica has seldom been reported. Cuevas *et al*.^10^ reported that the contact angle of liquid braze alloy on devitrified fused quartz decreased from 164° to approximately 100° by adding 1.5 wt% Ti to Ag-35 wt%Cu alloy, with a 5–10 min hold at 1123 K. Nevertheless, the result was still not satisfactory. The surface energy of the fused quartz was 0.3 J/m^2^ in the temperature range from 1100 to 1300 °C^11, 12^ compared with 1.85 J/m^2^ for SiC at 1430 °C^12^, indicating that the fused quartz possesses high surface chemical stability.

Recently, SiO_2f_/SiO_2_ composites have become a promising candidate as protection parts in recoverable satellite and carrier rockets due to their good thermal stability, low dielectric constant and good wave transmissivity^13, 14^. Unfortunately, similar to silica ceramic, the AgCuTi-SiO_2f_/SiO_2_ system also exhibits wettability issues. A study of the wetting of a SiO_2f_/SiO_2_ composite by AgCuTi alloy is still missing, which greatly limits the application of this promising ceramic composite.

In the past decade, carbon nanomaterials have been utilized to address the wetting issues because of their unique properties. Koltsov^15^ successfully reduced the contact angle of liquid Ni-Si alloy on alumina from 90° to 40° by depositing a carbon nanolayer on the alumina surface. Laha *et al*.^16^ found that β-SiC, an interfacial reaction product, could significantly improve the wettability between multi-walled carbon nanotubes and the Al-Si matrix. Recently, the edge of graphene has aroused many researchers’ interests due to its unique properties. It has been reported many times that the edge of graphene possesses high chemical reactivity, which was much higher than that of the corresponding inner carbons and nanotubes^17–20^. In addition, it has been stated that the decoration of functional groups tends to be preferentially located at the edge of graphene rather than in the bulk^21^, which further demonstrates the high chemical reactivity of the graphene edge. Most importantly, the reactivity of graphene nanosheets becomes stronger as the number of layers decrease, and a single graphene sheet has been shown to be 10 times more reactive than multilayer graphene^21^. Graphene containing 3–10 layers is identified as few-layer graphene^22^, which possesses similar physical and chemical properties as single-layer graphene. Meanwhile, the synthesis of vertical few-layer graphene VFG by the PECVD method has been reported over the past decade^23–27^, and a high consistency for the growth of VFG can be obtained. Therefore, we believe that the VFG grown by the PECVD method in this study with reactive edges will have an important impact on our wetting process.

Our previous work indicated that VFG can significantly promote the wetting of a SiO_2f_/SiO_2_ composite by AgCuTi liquid alloy^28^. However, the entire dynamic wetting process, as well as the mechanism of how the VFG promoted the wetting process, remains unclear. In this work, a VFG decorated SiO_2f_/SiO_2_ composite is developed using PECVD. The wettability of AgCuTi alloy on the surfaces of SiO_2f_/SiO_2_ composites with and without VFG decoration was evaluated using the sessile drop method. In particular, we thoroughly discuss the wetting mechanism of how VFG remarkably enhances the wetting of a SiO_2f_/SiO_2_ composite by AgCuTi alloy.

## Results and Discussion

### Characterization of VFG

**Figure 1:**
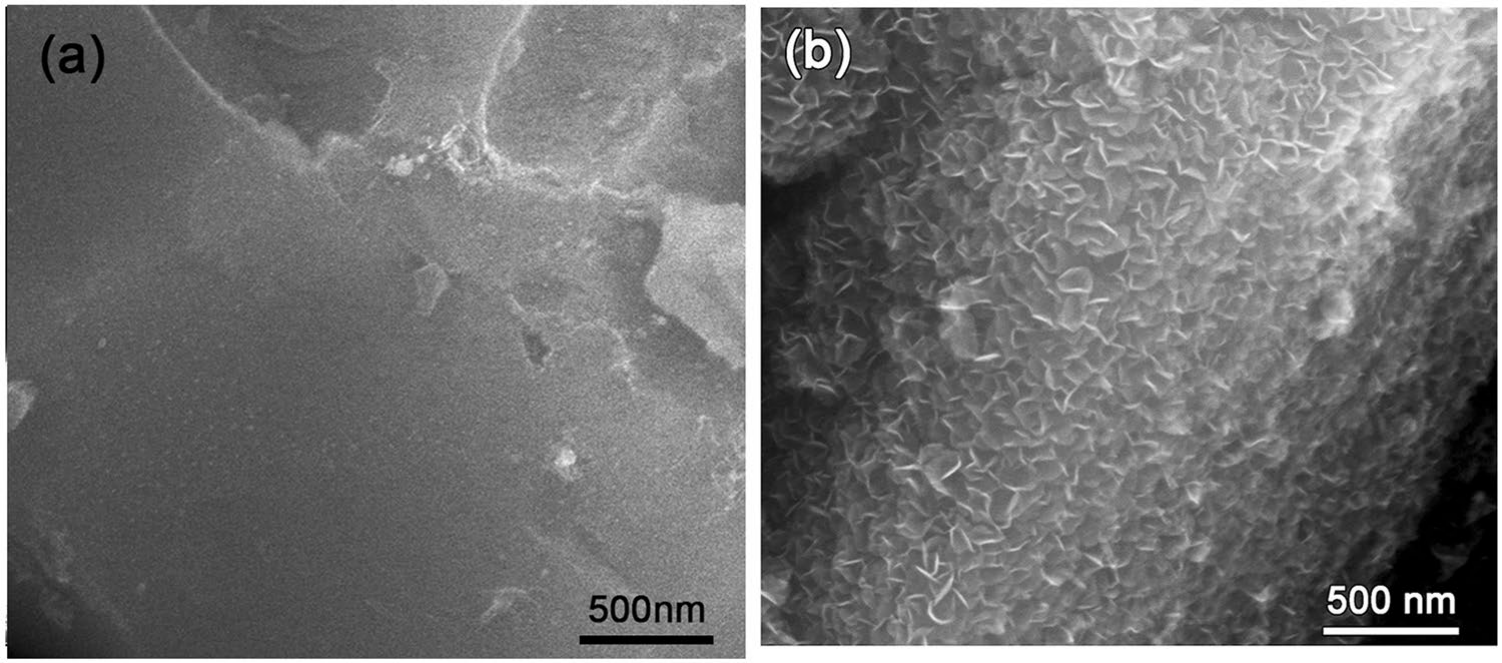
SEM images of (**a**) original SiO_2_ fibre without VFG decoration; (**b**) SiO_2_ fibre with VFG decoration.

VFG was firstly grown on the surface of the SiO_2f_/SiO_2_ composite using a PECVD method. To improve the growth efficiency of the VFG, a Cu(NO_3_)_2_ solution was selected as the catalyst suspension. The detailed synthesis process was stated in the “Method” section. Figure 1 illustrates the scanning electron microscopy (SEM) images for a SiO_2_ fibre with and without VFG decoration. It can be seen that the original SiO_2_ fibre exhibited a smooth surface, as shown in Fig. 1(a). In comparison, petal-like VFG was uniformly decorated on the surface of the SiO_2_ fibres after the PECVD treatment.

**Figure 2:**
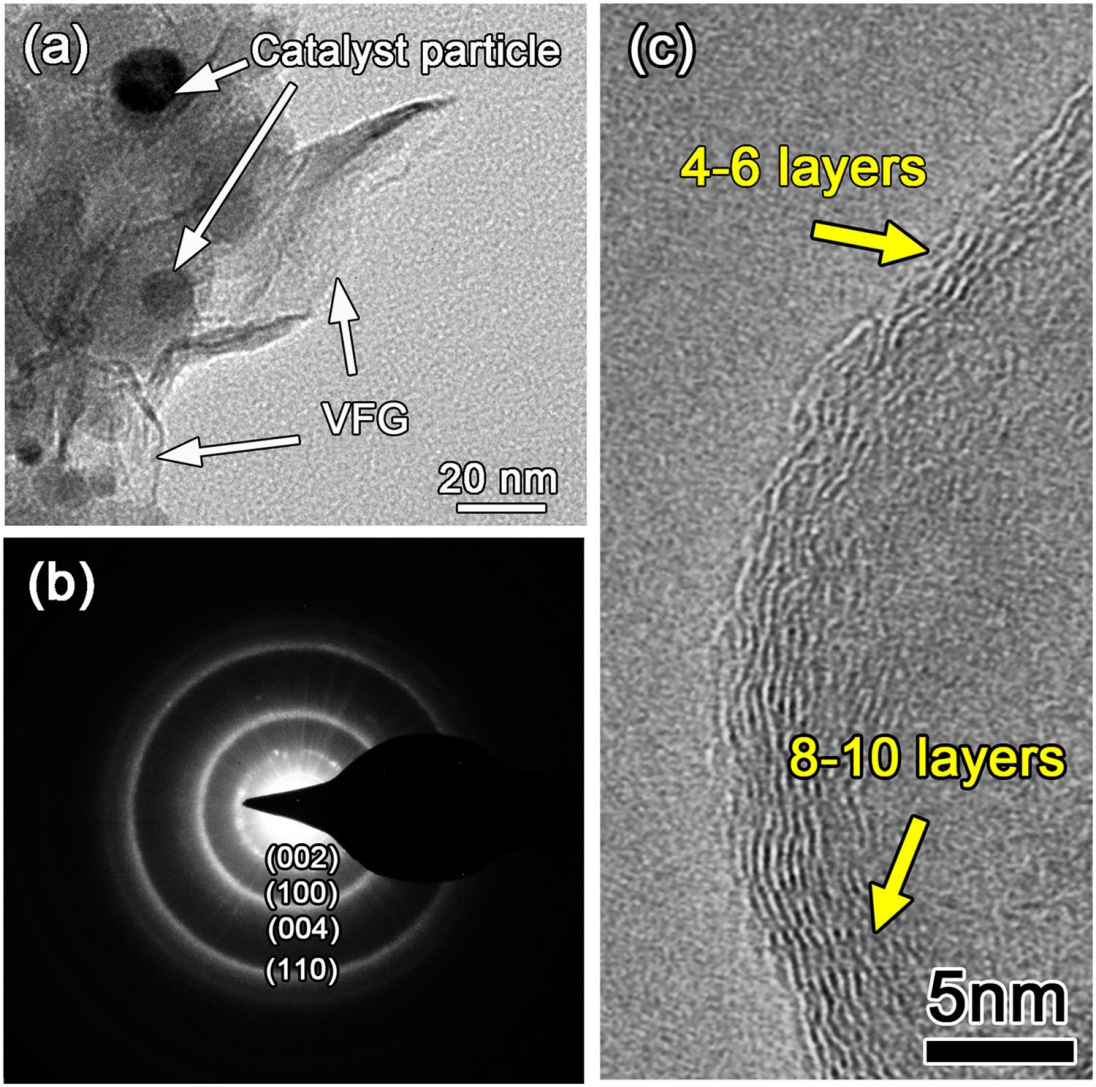
Characterization of the VFG. (**a**) TEM images of the VFG and the catalyst particles. The VFG was directly scratched from the surface of the SiO_2f_/SiO_2_ composite with VFG decoration. A carbon coated micro-grid was adopted to support the VFG. (**b**) Corresponding SAED pattern of the VFG. (**c**) Corresponding HRTEM image of the VFG.

To further characterize the fine structure of the VFG, transmission electron microscopy (TEM) together with HRTEM was employed. It can be seen from Fig. 2(a) that the VFG was so thin that its unfolded area was transparent. The black and grey particles were catalyst particles, which can be clearly seen through the transparent VFG. The selected area electron diffraction (SAED) pattern shown in Fig. 2(b) can be indexed as the polycrystalline graphite pattern. The HRTEM image of the VFG shown in Fig. 2(c) illustrates that the VFG close to the VFG/catalyst interface was relatively thick and was composed of 8–10 layers of graphene. In contrast, the edge of the VFG exposed outside was thinner and consisted of 4–6 layers of graphene, which was similar with the results obtained by Zhao^29^.

### Wetting phenomena of AgCuTi alloy on the SiO_2f_/SiO_2_ composites

**Figure 3:**
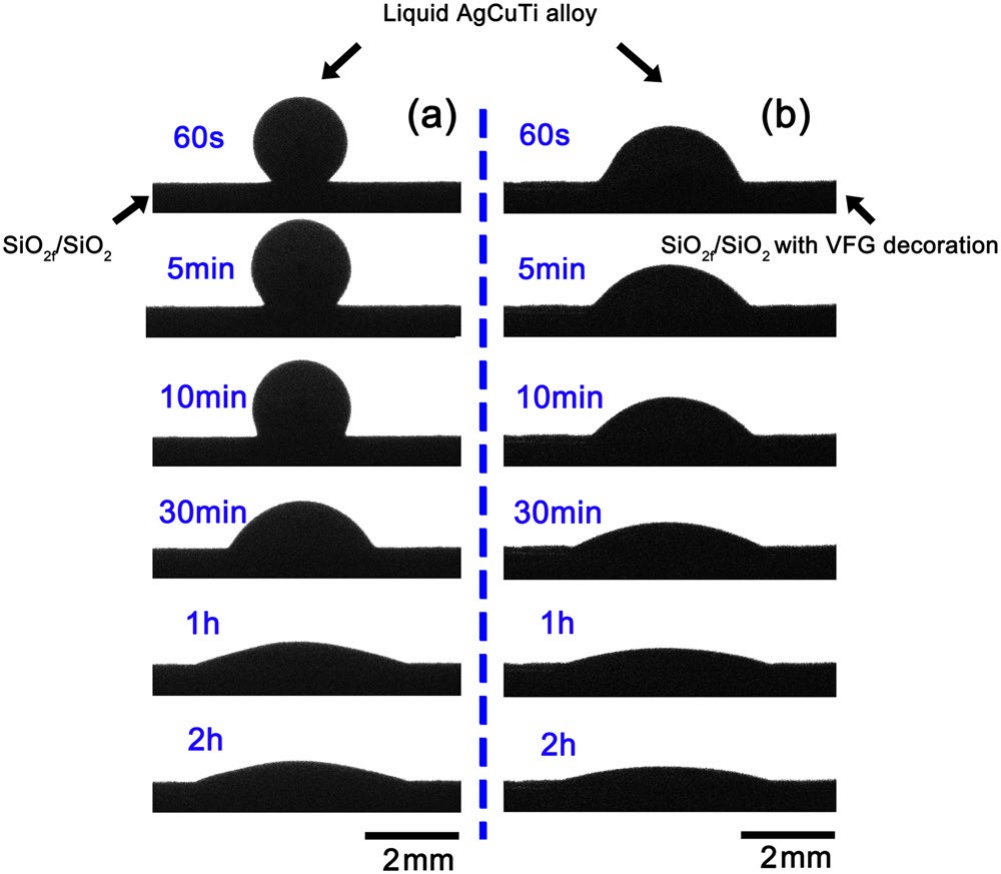
Captured images of the AgCuTi droplet on the surfaces of SiO_2f_/SiO_2_ composites: (**a**) without VFG decoration and (**b**) with VFG decoration.

To evaluate the wettability of the AgCuTi alloy on the surfaces of the SiO_2f_/SiO_2_ composites with and without VFG decoration, images of the wetting behaviour as a function of the holding time were obtained. The wetting experiments were conducted at 1123 K in vacuum. It can be seen from Fig. 3(a) that the AgCuTi alloy on the SiO_2f_/SiO_2_ composite without VFG decoration condenses into a ball, and only a small area at the bottom was in contact with the substrate at 60 s.

In comparison, the AgCuTi alloy already started spreading on the composite with the VFG decoration at 60 s. The AgCuTi alloy may spread and wet on the decorated substrate slowly when the alloy has not yet melted into liquid completely. As the holding time increased to 10 min, the contact angle of the AgCuTi alloy on the untreated composite was still higher than 90°. By contrast, the contact angle on the decorated substrate was significantly reduced under the same process parameters. Intriguingly, as the holding time was extended to 2 h, the AgCuTi alloy could wet the composites with and without the VFG decoration perfectly.

**Figure 4:**
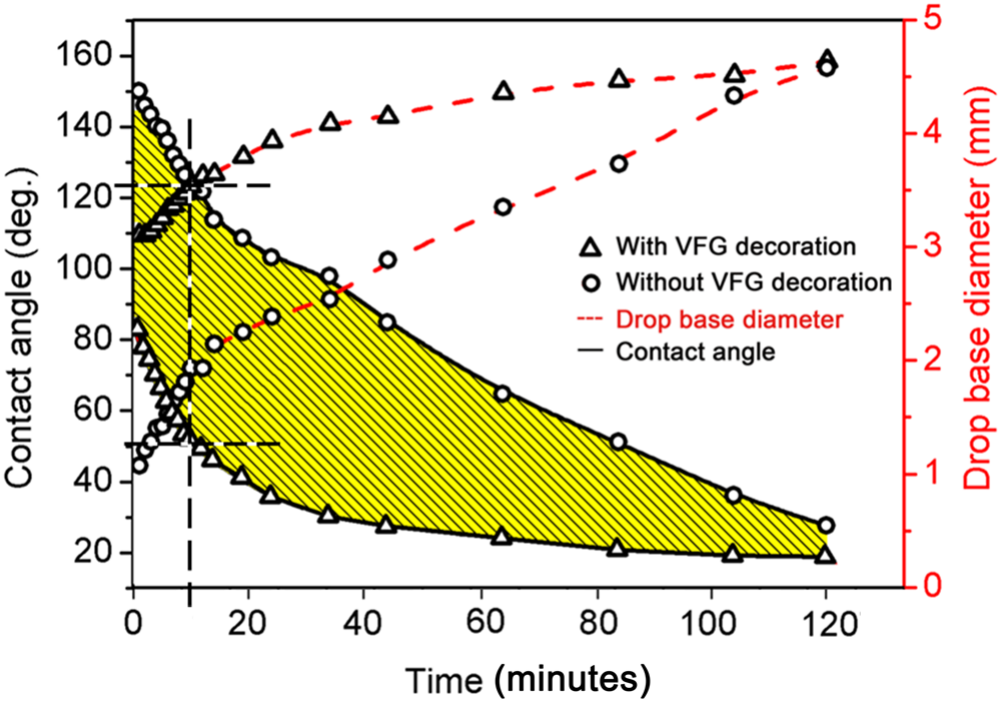
Contact angle (solid lines) and drop base diameter (dashed lines) as a function of time for AgCuTi alloy on SiO_2f_/SiO_2_ composites with and without VFG decoration.

Figure 4 shows the variation of the contact angle versus holding time for molten AgCuTi alloy on the SiO_2f_/SiO_2_ composites with and without VFG decoration at 1123 K in vacuum. The time origin was taken as the moment when the AgCuTi alloy melted completely.

It can be seen that the initial contact angle (drop base diameter) of the AgCuTi alloy on SiO_2f_/SiO_2_ composites with and without VFG decoration were 81° (3.1 mm) and 151° (1.2 mm), respectively. As our research system was a typical reactive wetting system, the spreading rate was mainly controlled by the growth of the reaction layer^30^. Due to the low surface energy of SiO_2_^11^, the chemical stability of the composite without VFG decoration would make the interfacial reaction relatively slow, leading to the slow spreading of the alloy. Thus, the base diameter of the drop was still small and the original contact angle of the AgCuTi alloy on the untreated SiO_2f_/SiO_2_ was very high. In comparison, after the AgCuTi alloy started to melt on the SiO_2f_/SiO_2_ composite with VFG decoration, increasingly more liquid filler formed and contacted with the VFG. The active VFG containing zigzag and armchair edges^19^ with high chemical reactivity would easily react with the AgCuTi alloy, resulting in the fast spreading of the alloy. Consequently, the original contact angle of the AgCuTi alloy on the SiO_2f_/SiO_2_ composite with the VFG decoration was much smaller, as shown in Fig. 4.

Afterwards, the contact angles of the two systems first declined rapidly until the holding time reached 15 min and then slowly dropped to a steady contact angle. The VFG mainly had effects on the initial spreading period of the AgCuTi alloy on the composite. Here, we take 10 min as an example. When the holding time reached 10 min, the contact angle of the AgCuTi alloy on the untreated substrate was still relatively high, at approximately 120°. By contrast, the contact angle of the AgCuTi alloy on the composite with the VFG decoration was approximately 50°, which was 60% lower than that of the untreated case. However, the diameter of the VFG layer was relatively small (tens of nanometres), which could consume only a small proportion of active Ti atoms. A large proportion of active Ti atoms would still react with the SiO_2f_/SiO_2_ composite, forming reliable joining. The work of adhesion *W*_ad_ when the holding time was 10 min can be expressed as follows:

1 $${W}_{{\rm{ad}}}=(1+\,\cos \,\theta )\gamma ,$$

where *γ* and *θ* are the surface tension of the liquid alloy and the contact angle between the liquid alloy and the SiO_2f_/SiO_2_ composite, respectively. Here, we can place 120° (without VFG decoration) and 50° (with VFG decoration) into equation (1), which estimates that *W*_ad_ with VFG decoration is 3.3 times greater than *W*_ad_ without VFG decoration. Therefore, it can be concluded that the SiO_2f_/SiO_2f_ composite with VFG decoration was better wetted by AgCuTi alloy and that joining with a higher work of adhesion could be obtained with a much shorter holding time than in the case without VFG decoration. However, it was interesting to observe that when the holding time was extended to 2 h, the AgCuTi alloy could well wet the SiO_2f_/SiO_2f_ composites with and without VFG decoration and the contact angles gradually became similar. This phenomenon will be discussed later in this paper.

### Interfacial reactions of the AgCuTi-SiO_2f_/SiO_2_ system with and without VFG decoration

Similar to other wetting systems that have been reported^8, 30^, the wetting of the SiO_2f_/SiO_2_ composites by the AgCuTi alloy in this paper was also reactive wetting. As a result, to investigate the wetting mechanism, it was particularly important to analyse the interfacial reaction layer.

**Figure 5:**
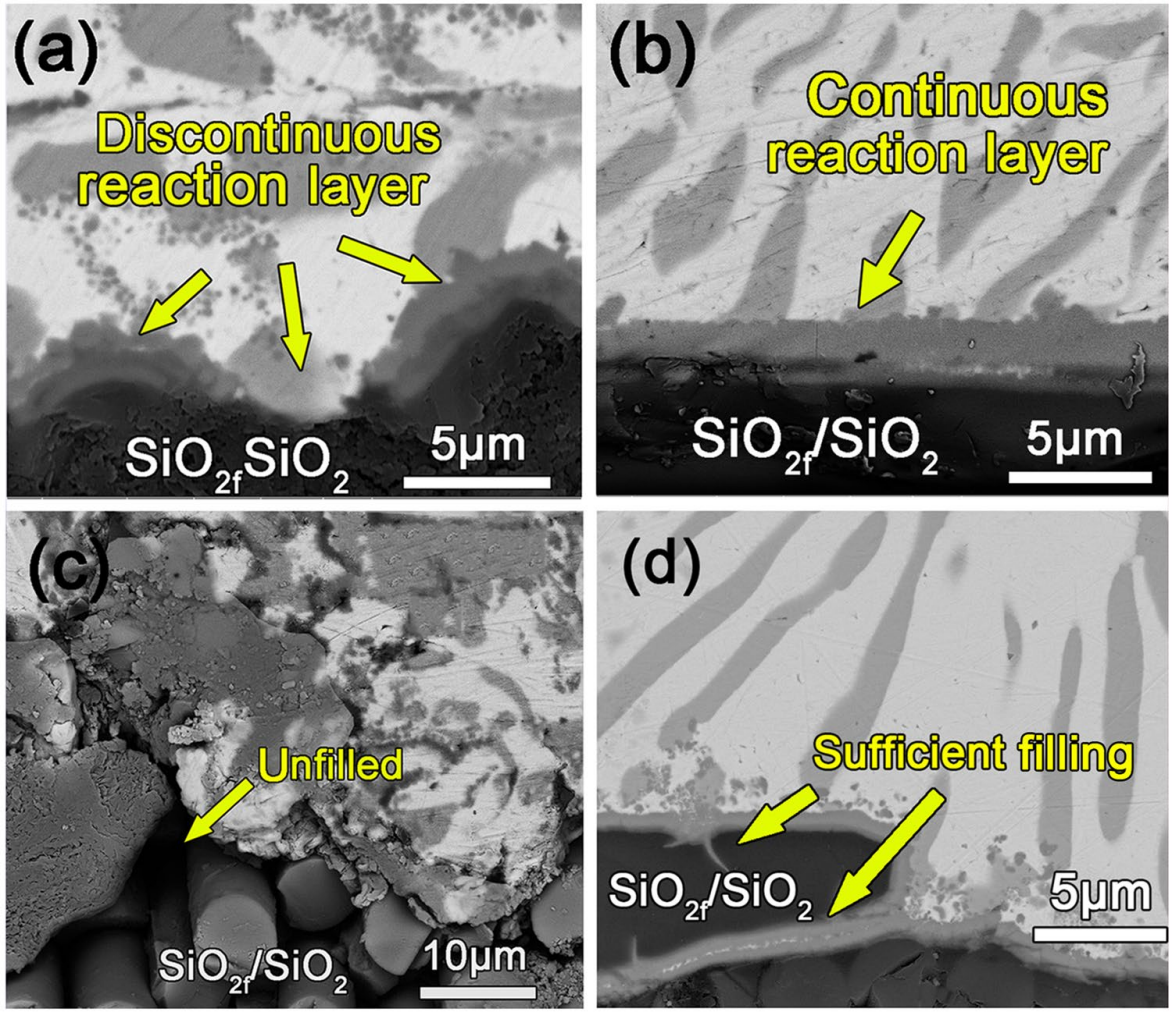
Microstructure of the AgCuTi-SiO_2f_/SiO_2_ wetting interface: (**a**,**c**) without VFG decoration; (**b**,**d**) with VFG decoration.

Figure 5 shows the SEM images of the microstructure of the AgCuTi-SiO_2f_/SiO_2_ wetting interface with and without the VFG decoration held at 1123 K for 10 min. 10 min was selected here which was proved to be the optimal time for the joining process in our previous results^28^. The interfacial reaction layers with and without the VFG decoration both consisted of double-layer reaction products. The layer adjacent to the SiO_2f_/SiO_2_ composite which was relatively thinner was mainly composed of Ti-Si and Ti-O compounds. The layer adjacent to the AgCu-4.5 wt.%Ti which was relatively thicker was mainly composed of a Cu-Ti-O phase. The EDS results of these two reaction layers were shown in Table 1. The results were similar with our previous study^28, 31^. It could be seen in Fig. 5(a) that part of the reaction layer without VFG decoration was discontinuous. By contrast, the reaction layer with VFG decoration was dense and continuous, as shown in Fig. 5(b). Due to the uneven surface morphology of the SiO_2f_/SiO_2f_ composites, different filling effects were obtained with and without VFG decoration.

**Table 1:** Composition (at%) of layers in Fig. 5.

	Ti	Ag	Cu	Si	O	Possible phase
Layer adjacent to AgCu-4.5 wt.%Ti	43.16	0.72	29.65	3.33	23.14	Cu-Ti-O
Layer adjacent to SiO_2f_/SiO_2_ composite	40.71	0.12	2.88	25.06	31.23	Ti-Si + Ti-O

For the interface without the VFG decoration, it was difficult for the AgCuTi alloy to fully fill the voids, as shown in Fig. 5(c). The contact and reaction between the braze filler and the SiO_2f_/SiO_2_ composite were relatively weak as a result. In comparison, after the VFG decoration, the braze filler readily filled the slits of the composite and reacted with the composite. Favourable contact and reaction between the braze filler and the composite were thus achieved. However, there was no difference in the composition of the reaction layer, which indicated that the effect of VFG on the reactive wetting process could hardly be observed from the SEM results.

### Interaction between Ti and VFG

As discussed above, the VFG could accelerate the wetting of the SiO_2f_/SiO_2_ composite by liquid AgCuTi alloy and promote the AgCuTi alloy to fully fill the gaps on the surface of the SiO_2f_/SiO_2_ composite. The VFG should be able to attract the Ti atoms. To confirm this conjecture and to investigate the interaction between VFG and Ti from a micro-perspective, VFG was grown on carbon-coated copper grids first. Then, Ti was deposited on the VFG using a resistance evaporation coating equipment. The reaction between Ti and VFG was characterized by HRTEM. Note that Ag and Cu atoms interacted weakly with the graphene, and chemical reactions seldom occurred in these systems^32–34^. Thus, combined with our wetting system, we focused on investigating the interaction between Ti and VFG and further confirmed the high chemical activity of the VFG from an experimental point of view.

**Figure 6:**
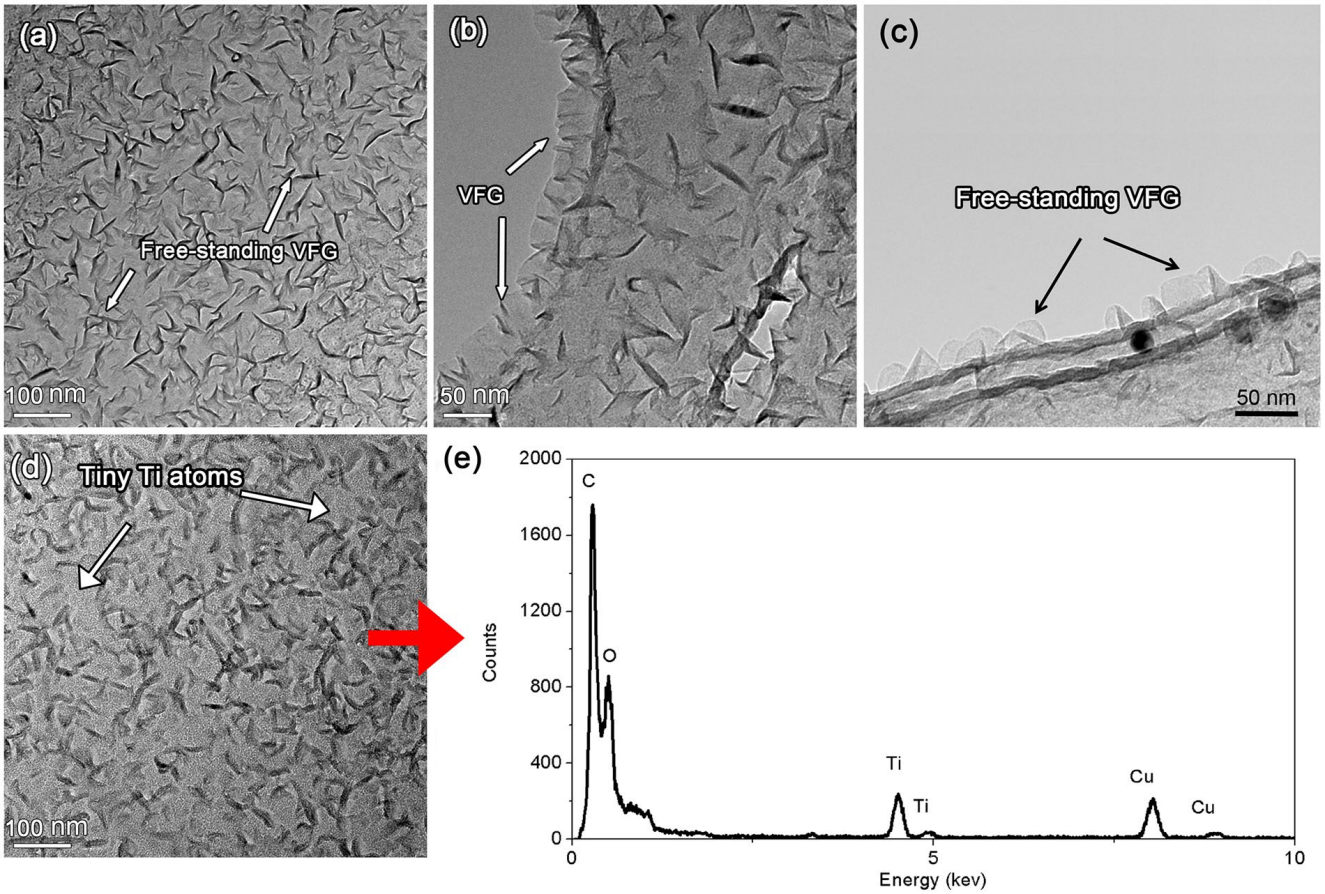
TEM image of VFG grown directly on a carbon coated micro-grid before and after the deposition of Ti: (**a,b**) and (**c**) before the deposition of Ti, (**d**) after the deposition of Ti, and (**e**) corresponding EDS result for the VFG after the deposition of Ti.

Figure 6(a,b) show the top-view TEM images of the VFG. The VFG was directly grown on the carbon coated copper micro-grid. Figure 6(b) shows that the VFG grown on the edge of the carbon film was sharp and transparent, indicating that the VFG was relatively thin. Figure 6(c) shows the side-view image of the VFG. It can be seen that the VFG was indeed vertically free-standing and the height was 50–100 nm. After the deposition of Ti atoms, numerous fine particles were attached to the surface of the VFG and the carbon film. The VFG maintained its original appearance, as shown in Fig. 6(d). The energy dispersive spectroscopy (EDS) results shown in Fig. 6(d) confirmed that Ti atoms had been successfully deposited on the surface of the VFG. Then, the samples were divided into two groups after the deposition of Ti atoms on the VFG. The VFG in the first group was only deposited with Ti without a subsequent heat treatment, which we called Sample A. The VFG in the other group was annealed at 600 °C for 1 min after the deposition of Ti, which we called Sample B. Then, HRTEM was employed to analyse the reaction between Ti and the VFG.

**Figure 7:**
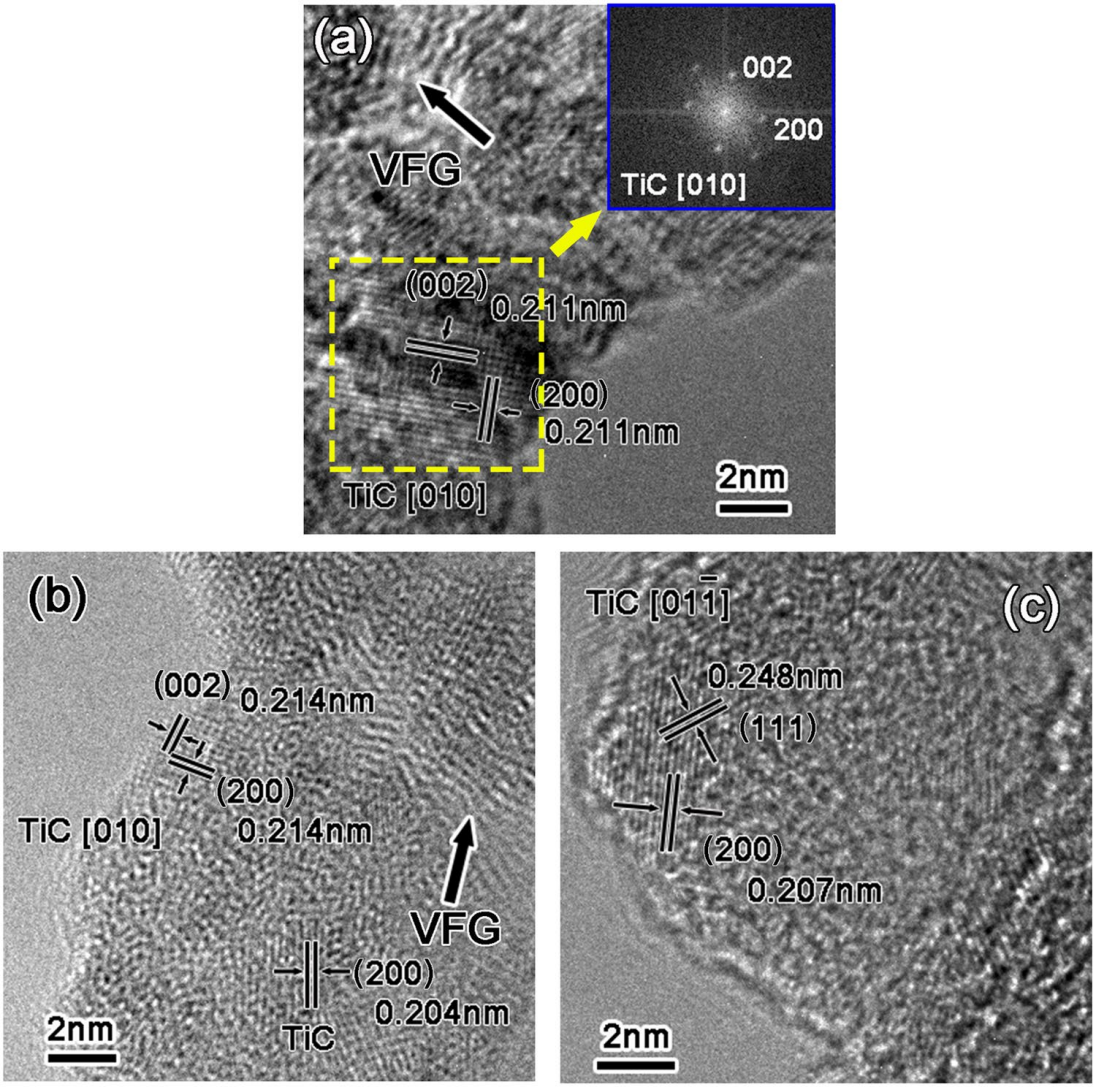
HRTEM images of the VFG after the deposition of Ti: (**a**) without subsequent heat treatment; (**b**,**c**) annealed at 600 °C for 1 min.

Figure 7(a) shows the HRTEM image of Sample A. It can be seen that the planes inside the square area bounded by the dashed line were perpendicular to each other. The spacing was measured to be 0.211 nm. According to the elements existing in our research system and the PDF card, the reaction product could be the TiC phase. The planes perpendicular to each other were (002) and (200). Fast Fourier Transformation was conducted in the region, as shown in Fig. 7(a), and the phase was further identified as TiC. The zone axis could be [010]. Additionally, there was still VFG remaining according to the HRTEM image. Thus, we can conclude that VFG was so reactive that it could react with Ti at room temperature.

Figures 7(b,c) show the HRTEM images for Sample B. In Fig. 7(b), TiC could also be detected. The planes perpendicular to each other were (002) and (200), and the corresponding spacing was 0.214 nm. A proportion of unreacted VFG could also be observed. In Fig. 7(c), the planes were (200) and (111) and the spacings were 0.207 nm and 0.248 nm, respectively. The corresponding angle was 54.74°.

Based on these observations, we can prove the existence of the TiC phase after the interaction between Ti and VFG. The HRTEM results verified that the VFG was so active that it could react with Ti, forming a TiC phase after annealing at 600 °C for 1 min or even at room temperature. In fact, the reason why VFG showed such high reactivity is mainly due to its reactive edges being exposed, as demonstrated by the experimental and theoretical experiments. It has been confirmed that the bond dissociation energy for the C-H bonds of the zigzag edge (2.86 eV) is much higher than that of carbon nanotubes (1.41 eV) and inner *sp* ^2^ hybridized carbons (0.83 eV)^20^, demonstrating the high reactivity of the exposed edges. Meanwhile, defects were inevitably created during the PECVD process, and the curvature caused by the basal plane fluctuation on VFG would also become potential reactive spots that could enhance the reactivity of the VFG^20, 35^. The relatively high chemical reactivity of the VFG and its reaction with Ti promoted the wetting of the SiO_2f_/SiO_2_ composite with VFG decoration by the AgCuTi alloy.

**Figure 8:**
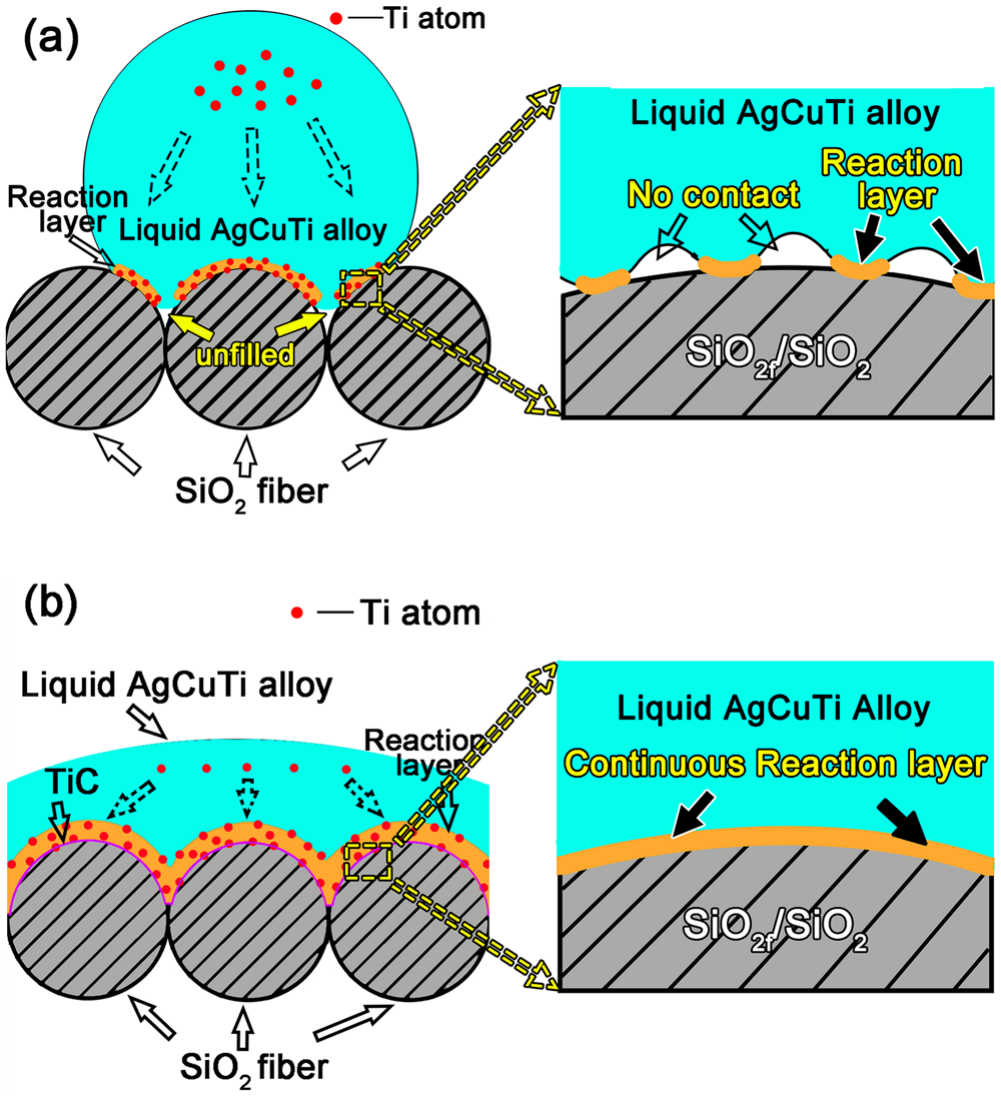
Illustration of the wetting of a SiO_2f_/SiO_2_ composite by AgCuTi liquid alloy at 850 °C for 10 min: (**a**) without VFG decoration and (**b**) with VFG decoration.

Figure 8 shows the wetting mechanism before and after the VFG decoration. When the surface of the SiO_2f_/SiO_2_ composite was not decorated with VFG, the AgCuTi alloy reacted slowly with the SiO_2_ fibres due to their high chemical stability.

It was noteworthy that the liquid AgCuTi alloy could still not completely fill the gaps and voids between the SiO_2_ fibres, which could be easily observed in the SEM images of the interface, as shown in Fig. 5(c). In addition, from the magnified schematic illustration of the AgCuTi-SiO_2f_/SiO_2_ interface without the VFG decoration shown in Fig. 8(a), the liquid AgCuTi alloy could not achieve full contact with the surface of the SiO_2_ fibre, which would lead to a partial metallurgical reaction at the AgCuTi-SiO_2f_/SiO_2_ interface. The reaction layer was distributed discontinuously, which meant that the liquid alloy spread on a wettable reaction layer combined with a proportion of the non-wettable SiO_2_ fibre surface. The corresponding wetting process was relatively slow and difficult.

As for the case with the VFG decoration shown in Fig. 8(b), due to the high chemical reactivity of the VFG, the active Ti in the AgCuTi alloy readily reacted with the VFG, forming a small proportion of TiC phases in the interfacial zone. Then, the frontier of the three-phase line moved forward, and the gaps were filled perfectly because the TiC phase has a metallic character^36, 37^ and was therefore more compatible with the AgCuTi alloy and more wettable by the liquid AgCuTi alloy than the original SiO_2_ fibres. More importantly, the liquid alloy could achieve substantial contact with the SiO_2_ fibre at the microscopic level; therefore, a dense and continuous reaction layer could be obtained, as shown in Fig. 8(b). The contact angle *θ*_*Cas*_ on a non-homogeneous surface is defined by Cassie^38^ as follows:

2 $$\cos \,{\theta }_{Cas}={{\Phi }}_{TiC}\,\cos \,{\theta }_{1}+(1-{{\Phi }}_{TiC})\cos \,{\theta }_{2}$$

where *Φ*_*TiC*_ is the surface fraction of TiC near the triple line. *θ*_*1*_ and *θ*_*2*_ are the equilibrium contact angles of the AgCuTi alloy on the titanium carbide and SiO_2f_/SiO_2_ composite, respectively. In our study, the contact angles with and without VFG were *θ*_*Cas*_ and *θ*_*2*_, respectively (*θ*_*Cas*_ = *θ*_*1*_ if *Φ*_*TiC*_ = 1). It can be easily concluded that cos *θ*_*2*_ < cos *θ*_*Cas*_ ≤ cos *θ*_*1*_ if 0 < *Φ*_*TiC*_ ≤ 1, which led to *θ*_*2*_ > *θ*_*Cas*_ as a result. When *Φ*_*TiC*_ approached 1, *θ*_*Cas*_was equal to *θ*_*1*_ and thus the contact angle for the case with the VFG decoration decreased. Due to the small size of the VFG in our wetting system, only a slight proportion of Ti would be consumed. The residual Ti atoms would subsequently react with the SiO_2_ fibres. Therefore, the existence of the VFG only affected the wettability of the AgCuTi alloy on the composite in the initial wetting stage. When the holding time was extended to sufficiently long (such as 2 hours), a dense and continuous Cu-Ti-O reaction layer could form for both cases with and without the VFG decoration. The Cu-Ti-O phase, with its metallic characteristics, could be perfectly wetted by the AgCuTi alloy^8^. Therefore, the final contact angles (with and without VFG decoration) were both relatively small and showed no significant difference. The surface roughness is another key important factor that should be taken into consideration during the wetting process. However, as the wetting system in this paper was reactive wetting, the VFG would react with the Ti from liquid AgCuTi alloy. As a result, the surface morphology of the SiO_2f_/SiO_2_ composite with the VFG decoration will not maintain its initial morphology during the wetting stage. So the initial surface roughness of the SiO_2f_/SiO_2_ composite with the VFG decoration will have little impact on the reactive wetting process compared with the case without the VFG decoration.

## Conclusions

In this study, VFG was fabricated on the surface of a SiO_2f_/SiO_2_ composite using the PECVD technique. Compared to the case without VFG decoration, the liquid AgCuTi alloy spread much faster and showed better wettability on the surface of the SiO_2f_/SiO_2_ composite with VFG decoration in the initial wetting stage. The contact angle of the AgCuTi alloy on the SiO_2f_/SiO_2_ composite with VFG decoration was approximately 50°, which was 60% lower than that of the untreated case. Meanwhile, the reaction layer became more dense and continuous without unfilled voids. The VFG with reactive edges possessed such a high chemical reactivity that it could easily react with the element Ti after the annealing process or even at room temperature. Thus, fast spreading of the liquid AgCuTi alloy was promoted on the SiO_2f_/SiO_2_ composite in the initial stage of the wetting process. Then, the residual active liquid braze alloy reacted with the SiO_2_ fibres and wetted on the wettable Cu-Ti-O reaction layer in place of the original non-wettable SiO_2_ fibres until a final steady state was reached. The observed effect of VFG on promoting the wetting of SiO_2f_/SiO_2_ composites by AgCuTi alloy laid a solid foundation for the subsequent joining process.

## Methods

The original SiO_2f_/SiO_2_ composite was synthesized by the immersion of quartz fibres braided at two dimensions in silica sol and the fibres were then sintered. The composite was obtained from Institute for Advanced Ceramics, Harbin Institute of Technology.The VFG on the surface of the SiO_2f_/SiO_2_ composite was synthesized by the PECVD method. Before the PECVD process, the SiO_2f_/SiO_2_ composite was immersed into 20 mL of homogenized Cu(NO_3_)_2_ solution (0.1 mol/L) for 5 min. Then, the SiO_2f_/SiO_2_ composite was taken out, dried in air for a few minutes and kept in the radio frequency (13.56 MHz) PECVD reaction chamber at 800 °C in a mixture of CH_4_/Ar = 20 sccm:80 sccm. The furnace pressure, RF (radio frequency) time and RF power were kept at 6 Torr, 60 min and 200 W, respectively. The unit model of the PECVD equipment is JGP-400A provided by Shenyang Scientific Instrument Development Centre, Chinese Academy of Sciences. The equipment is composed of 4 parts: Vacuum pumping part, Heating part, Chamber and Radio frequency part.

The sessile drop method was selected for the wetting experiments at 1123 K. The SiO_2f_/SiO_2_ composite (10 mm × 10 mm × 2 mm) was placed on an alumina supporter inside a stainless chamber and adjusted to the horizontal position. An AgCu-4.5 wt.%Ti foil was selected as the braze alloy (100 μm thick, 5 mm × 10 mm) which was curled and put on a SiO_2f_/SiO_2_ composite substrate. The melting temperature of the AgCu-4.5 wt.%Ti foil was round 1053 K. Then, the furnace chamber was evacuated to 2.0 × 10^−6 ^mbar. The composite was heated up to 1123 K at a rate of 20 Kmin^−1^, and the temperature was held until a stable contact angle was achieved. The contact angles of the AgCu-4.5 wt.%Ti alloy on SiO_2f_/SiO_2_ composites with and without VFG decoration were calculated from the droplet images using the OCA20 software produced by Dataphysics Company in Germany. The software is capable of measuring the drop base radius R and drop contact angle θ within 1% accuracy.

Morphologies and elemental compositions of the interfacial reaction products were examined using a SEM (Helios Nanolab600i scanning electron microscope) equipped with an energy dispersive spectrometer (EDS). Transmission electron microscopy (TEM, Tecnai G2 F30) together with the HRTEM method was employed to analyse the fine microstructure of the reaction products.
